# Validation of the GALAD model for diagnosing HBV-related hepatocellular carcinoma in Chinese patients

**DOI:** 10.1016/j.clinsp.2026.100882

**Published:** 2026-02-17

**Authors:** KeCheng Li, Fei Xia, XiaoYa Wu

**Affiliations:** Laboratory Department of Ruian People's Hospital, Zhejiang Province, China

**Keywords:** HCC, GALAD, AFP, AFP-L3%, DCP

## Abstract

**Background:**

The GALAD model integrates serological biomarkers (AFP, AFP-L3%, DCP) with demographic factors to estimate the presence of Hepatocellular Carcinoma (HCC). However, its applicability in Chinese populations with HBV-related HCC remains underexplored. This study validated the clinical utility of GALAD in this specific population.

**Methods:**

A retrospective cohort study enrolled 217 HBV-related HCC patients, 210 patients with benign Liver disease without cirrhosis, 247 Cirrhosis patients, and 220 healthy controls. Serum levels of AFP, AFP-L3%, and DCP were measured. Receiver Operating Characteristic (ROC) curve analysis assessed diagnostic performance, and the Hosmer-Lemeshow test evaluated model calibration. Serum levels of AFP, AFP-L3%, and DCP were measured. Receiver Operating Characteristic (ROC) curve analysis assessed diagnostic performance, and the Hosmer-Lemeshow test evaluated model calibration.

**Results:**

GALAD demonstrated superior diagnostic performance, with an Area Under the Curve (AUC) of 0.942. At an optimal cutoff of 1.89, sensitivity and specificity were 89.91 % and 81.51 %, respectively. The model showed excellent calibration (χ^2^ = 8.934, p > 0.05), confirming its reliability for Chinese HBV-related HCC patients.

**Conclusion:**

The GALAD model outperforms individual biomarkers in diagnosing HBV-related HCC in the Chinese population, highlighting its potential utility in clinical practice. This cost-effective tool holds particular promise for the tertiary prevention of HBV-related HCC, especially in resource-limited settings.

## Highlights

• GALAD model shows high accuracy in diagnosing HBV-related HCC in Chinese patients.
• GALAD outperforms AFP, AFP-L3 %, and DCP in diagnostic performance.
• GALAD is a cost-effective tool for HCC screening in HBV-endemic and in resource-limited regions.

## Introduction

Hepatocellular Carcinoma (HCC) is the most prevalent liver malignancy globally and significantly contributes to digestive system cancers. Its aggressive nature and poor outcomes in late stages necessitate early diagnosis. Thus, developing cost-effective serological screening methods for early detection is crucial for improving patient prognoses.

In 2013, Johnson et al. introduced the GALAD (Gender, Age, Lens culinaris agglutinin reactive AFP, AFP, Des-γ-carboxy prothrombin) model, a diagnostic tool integrating three serological markers (AFP, AFP-L3%, DCP) with patient gender and age.^1^ This model has garnered recognition for its high diagnostic accuracy in Western HCC populations,^2^, ^3^, ^4^, ^5^, ^6^ where alcohol-related liver disease,as well as HBV subtype A and D, are predominant.^7^^,^^8^ However, considering that the primary causes of HCC in China are infections with HBV (HBV), particularly HBV subtypes B and C,^9^ the applicability of GALAD in this population remains to be rigorously validated.

This study aims to bridge this gap in knowledge by examining the diagnostic value and clinical applicability of GALAD in the Chinese HBV-related HCC patient cohort, with the ultimate goal of enhancing early detection strategies and improving patient outcomes.

## Materials and methods

### Study subjects

HBV-related HCC Group: 217 patients diagnosed with HCC at Ruian People’s Hospital (November 2019–October 2021), HBsAg-positive, excluding HCV, NAFLD, alcoholic liver disease, drug-induced liver damage, or other liver diseases. Male-to-female ratio: 160:57; mean age: 55.94 ± 10.6 years.

Cirrhosis Group: 247 patients with liver cirrhosis, excluding HCC, alcoholic liver disease, drug-induced liver damage, or other malignancies. Male-to-female ratio: 178:69; mean age: 57.3 ± 11.3 years.

Healthy Control Group: 220 individuals without liver or systemic diseases. Male-to-female ratio: 151:69; mean age: 56.51 ± 13.7 years.

This study was conducted in accordance with the Declaration of Helsinki and approved by the Ethics Committee of Ruian People's Hospital (Approval n° LZM2021056). Given the retrospective nature of the study and the use of anonymized data, the requirement for individual informed consent was waived by the Ethics Committee.

This diagnostic study was conducted and reported in accordance with the STARD (Standards for Reporting of Diagnostic Accuracy Studies) guidelines.

### Study methods

#### Serological marker detection methodology

AFP levels were measured using electrochemiluminescence, executed on a dedicated analyzer equipped with manufacturer-specified reagents to ensure accuracy and precision. The proportion of Lens culinaris agglutinin-reactive AFP (AFP-L3%) was determined through the affinity centrifugal adsorption method, employing commercial reagent kits sourced from a reputable supplier to guarantee the reliability of the assay. Lastly, Des-γ-Carboxy Prothrombin (DCP) concentrations were assessed using a proprietary methodology on a fully automated immunoassay analyzer, complemented by matching detection reagents to maintain the highest standards of analysis.

AFP, AFP-L3%, and DCP were measured simultaneously as a complete diagnostic panel in all enrolled patients; no missing biomarker data were present in the final analysis.

#### GALAD model calculation formula [1]

Z=−10.08+0.09×Age+1.67×Gender+2.34×log10(AFP)+0.04×AFP−L3%+1.33×log10(DCP).Genderwascodedasmale=1andfemale=0.

### Statistics analysis

All statistical analyses and visualizations were performed using *R* (version 4.4.1, *R* Foundation for Statistical Computing, Vienna, Austria). Categorical variables were expressed as percentages and compared across groups using chi-square tests (chisq.test function, stats package). To address non-normality in continuous variables, logarithmic transformations were applied to AFP and DCP values. Non-normally distributed continuous variables were reported as medians with Interquartile Ranges (IQRs), and group comparisons were conducted using the Kruskal-Wallis H test (kruskal.test function, stats package), with the Bonferroni correction applied to control for Type I error inflation due to multiple comparisons.

Receiver Operating Characteristic (ROC) curves were generated using the pROC package, and Area Under the Curve (AUC) comparisons were performed using DeLong’s test (roc.test function, pROC package) to assess diagnostic performance. The goodness-of-fit of the GALAD model was evaluated using the Hosmer-Lemeshow test via the Resource Selection package, stratifying patients into 10-risk deciles to verify model calibration.

All plots, including boxplots with jittered points (beeswarm), ROC curves, and calibration plots, were generated using the ggplot2, ggbeeswarm, and gridExtra packages, ensuring high-quality, publication-ready visualizations. Statistical significance was defined as p < 0.05.^10^

## Results

### Biomarker levels across study groups

**Fig. 1:**
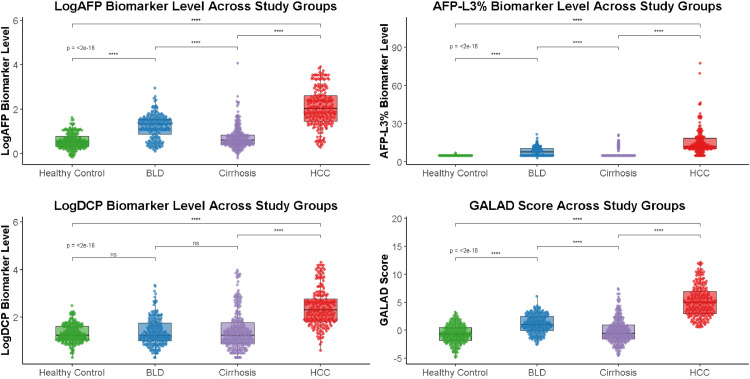
Jittered points of biomarker levels across study groups.

Significant differences were observed in AFP, DCP, their logarithmic transformations (LogAFP and LogDCP), AFP-L3%, and GALAD scores across the four study groups (healthy controls, benign liver disease, cirrhosis, and HBV-related HCC; all p < 0.05). Consistent with the overall analysis, the HCC group demonstrated markedly higher levels of all biomarkers compared with the other three groups. In addition, sex-stratified analyses (Table 1) revealed similar patterns in both males and females, with HCC patients showing the highest values across biomarkers. The detailed distribution of biomarker levels is presented in Table 1 and Fig. 1.

**Table 1:** Biomarker comparison across three participant groups.

**Variable**	**Healthy Control Group** **(n = 220)**	**BLD Group** **(n = 210)**	**Cirrhosis Group** **(n = 247)**	**HBV-related HCC Group (n = 217)**	**Statistical value**	**p**
AFP (ng/mL)	3.2 (2.1∼6.1)	21.1 (7.1∼35.8)	4.1 (2.7∼7.5)	108 (28.6∼430.6)	431.7	<0.05
Male	3.5 (2.3∼6.5)	16.6 (5.1∼34.0)	3.9 (2.6∼6.7)	94.4 (26.9∼755.7)	294.5	<0.05
Female	2.7 (1.8∼4.5)	22.0 (12.9∼42.3)	4.3 (2.6∼9.3)	132 (43.4∼300.0)	140.8	<0.05
Log_10_AFP	0.5 (0.3∼0.8)	1.3 (0.85∼1.55)	0.6 (0.4∼0.8)	2.0 (1.5∼2.6)	431.7	<0.05
Male	0.5 (0.4∼0.8)	1.2 (0.7∼1.5)	0.6 (0.4∼1.0)	2.1 (1.4∼2.9)	294.5	<0.05
Female	0.4 (0.3∼0.7)	1.3 (1.1∼1.6)	0.6 (0.4∼1.0)	2.1 (1.6∼2.5)	140.8	<0.05
AFP-L3%	5.0 (5.0∼5.0)	7.8 (5.0∼10.6)	5.0 (5.0∼5.0)	12.0 (10.3∼18.5)	457.0	<0.05
Male	5.0 (5.0∼5.0)	7.2 (5.0∼10.5)	5.0 (5.0∼5.0)	11.8 (10.0∼18.0)	334.8	<0.05
Female	5.0 (5.0∼5.0)	8.0 (5.0∼10.5)	5.0 (5.0∼5.0)	12.2 (11.0∼19.2)	127.9	<0.05
DCP (mAU/mL)	17.0 (11.0∼40.8)	17.0 (10.0∼56.0)	19.0 (8.0∼64.0)	198.0 (68.00∼593.5)	261.2	<0.05
Male	17.0 (11.0∼40.0)	16.0 (9.0∼40.0)	16.0 (8.0∼51.5)	178.0 (60∼744.8)	184.7	<0.05
Female	17.0 (10.0∼45.5)	28.0 (10.0∼89.0)	23.0 (8.2∼70.8)	203.0 (126.00∼522.5)	86.0	<0.05
Log_10_DCP	1.2 (1.0∼1.6)	1.2 (1.0∼1.75)	1.3 (0.9∼1.8)	2.3 (1.8∼2.8)	261.2	<0.05
Male	1.2 (1.0∼1.6)	1.2 (1.0∼1.6)	1.2 (0.9∼1.7)	2.2 (1.8∼2.9)	184.7	<0.05
Female	1.2 (1.0∼1.7)	1.4 (1.0∼1.9)	1.4 (0.9∼1.8)	2.3 (2.1∼2.7)	86.0	<0.05
GALAD	-0.8 (-2.0∼0.3)	0.7 (-0.3∼2.2)	-0.4 (-1.4∼1.0)	4.4 (2.5∼6.3)	448.6	<0.05
Male	-0.2 (-1.0∼0.8)	1.2 (0.2∼2.7)	-0.5 (-1.4∼0.8)	5.1 (3.3∼7.5)	323.2	<0.05
Female	-2.1 (-3.0∼1.5)	0.6 (-0.4∼1.9)	-0.4 (-2.2∼1.3)	4.1 (2.6∼5.7)	147.3	<0.05

**Note:** Data are presented as median (Interquartile Range, IQR).

### Diagnostic efficacy analysis of AFP, AFP-L3%, DCP, and GALAD scores for HBV-related HCC

**Fig. 2:**
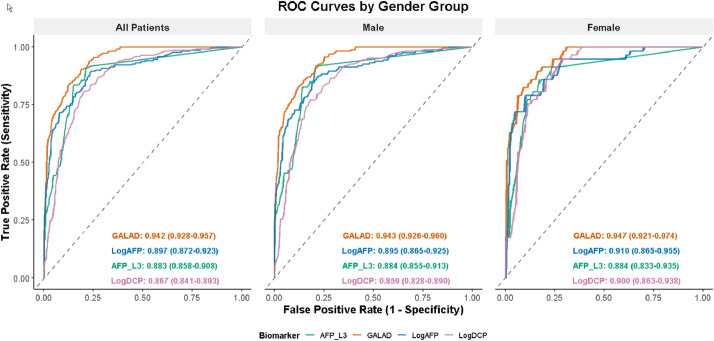
Comparison of ROC curves for each indicator in diagnosing HCC.

Comparative analysis of diagnostic performance showed that the GALAD score achieved the highest discriminatory ability, with an overall AUC of 0.942, outperforming individual biomarkers including LogAFP (AUC = 0.897), AFP-L3% (AUC = 0.883), and LogDCP (AUC = 0.867). In sex-stratified analyses (Table 2), GALAD remained superior in both males (AUC = 0.926) and females (AUC = 0.947), with slightly higher specificity observed in females and higher sensitivity in males. These findings indicate that the advantage of GALAD over individual biomarkers is consistent across sexes. The detailed ROC results are provided in Table 2 and illustrated in Fig. 2.

**Table 2:** Diagnostic efficacy comparison of biomarkers in HBV-related HCC vs. Non-HCC groups.

**Variable**	**AUC**	**95 % CI**	**Cut Off**	**Youden Index**	**Susceptibility**	**Specificity**
LogAFP	0.897	0.872‒0.922	1.12	0.66	89.5	76.3
Male	0.895	0.865‒0.925	1.23	0.66	85.7	80.0
Female	0.910	0.865‒0.955	1.63	0.69	78.9	90.1
AFPL3	0.883	0.856‒0.907	7.05	0.70	90.8	78.8
Male	0.884	0.855‒0.913	7.0	0.70	90.7	79.6
Female	0.884	0.833‒0.935	9.40	0.69	86.0	82.8
LogDCP	0.867	0.840‒0.891	1.76	0.61	80.7	79.8
Male	0.859	0.828‒0.890	1.76	0.58	77.0	81.8
Female	0.900	0.863‒0.938	1.69	0.61	94.7	71.4
Galad	0.942	0.928‒0.957	1.89	0.71	89.9	81.5
Male	0.926	0.926‒0.960	1.84	0.71	91.9	79.2
Female	0.947	0.921‒0.974	2.3	0.74	82.5	91.1

### Assessment and validation of calibration capability for the GALAD prediction model

**Fig. 3:**
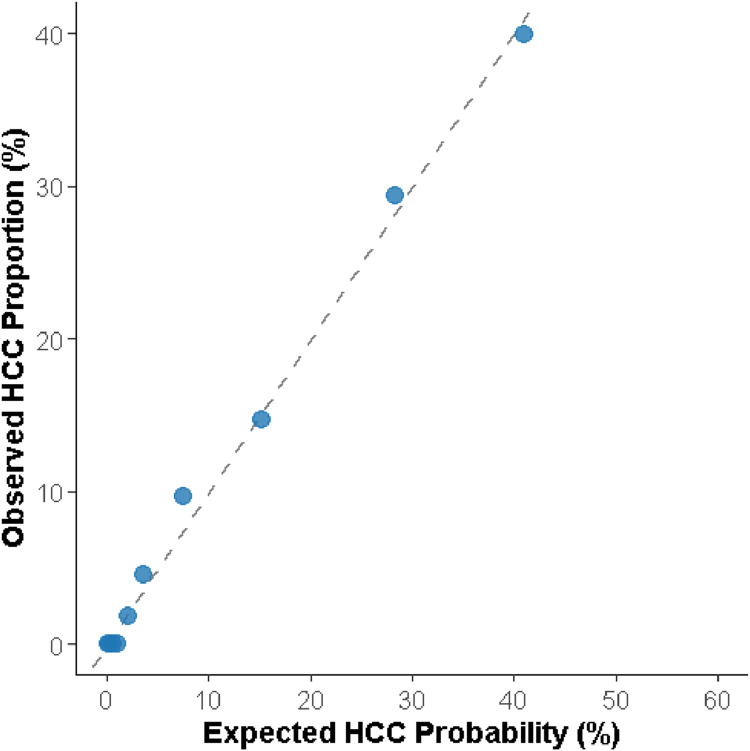
Goodness of fit test of GALAD model.

To evaluate the calibration performance of the GALAD prediction model, the authors performed external validation using an independent cohort dataset from our institution. The Hosmer-Lemeshow goodness-of-fit test was applied to assess the agreement between predicted and observed probabilities. The test result (χ^2^ = 8.934, p > 0.05) indicated no significant deviation from the model's predictions, suggesting good calibration. Detailed results are presented in Table 3 and Fig. 3.

**Table 3:** Contingency table utilized in Hosmer-Lemeshow goodness-of-fit tests.

**Step**	**NO HCC**		**HBV-related HCC**	
	**Measurement**	**Expected**	**Measurement**	**Expected**
1	89	88.751	0	0.249
2	89	88.306	0	0.694
3	89	87.741	0	1.259
4	89	86.674	0	2.326
5	85	84.499	4	4.501
6	79	81.000	10	8.000
7	68	72.424	21	16.576
8	57	55.847	32	33.153
9	25	27.289	64	61.711
10	6	3.469	87	89.531

## Discussion

This study validates the GALAD model’s diagnostic accuracy for HBV-related HCC in Chinese patients, achieving an AUC of 0.942, sensitivity of 89.91 %, and specificity of 81.51 %, consistent with global studies despite differing etiologies (HBV subtypes B and C in China vs. NAFLD, HCV, and HBV subtypes A and D in Western populations).^5^^,^^11^, ^12^, ^13^ Notably, the GALAD score achieved an area under the receiver operating characteristic curve (AUC) of 0.942, outperforming individual biomarkers such as LogDCP, LogAFP, and AFP-L3% in the HBV-positive cohort, highlighting the value of a multi-biomarker approach in enhancing diagnostic accuracy.^13^, ^14^, ^15^, ^16^

The GALAD score has been extensively validated across diverse populations in Europe, Asia, the Americas, and Africa, consistently demonstrating robust diagnostic performance.^3^^,^^17^^,^^18^ A 2023 systematic review analyzing data from 15 studies reported a pooled sensitivity of 82 %, specificity of 89 %, and AUC of 0.92.^7^ Specific regional studies further corroborate its utility: in a European cohort with alcohol-related HCC, the GALAD score achieved an AUC of 0.9, with sensitivity of 81.2 % and specificity of 85.5 %^19^; in a U.S. cohort predominantly linked to HCV and alcohol misuse, it demonstrated sensitivity, specificity, and AUC values of 71.4 %, 78.5 %, and 0.84, respectively^20^; and in an African cohort with HV-related HCC, the corresponding metrics were 81 %, 86 %, and 0.87 %.^21^ In contrast, the present study reported higher diagnostic metrics (sensitivity: 89.9 %; specificity: 81.5 %; AUC: 94.2 %), This enhanced performance can be attributed to distinct cohort characteristics. Firstly, the inclusion of controls without underlying liver disease improved specificity. More fundamentally, the predominance of HBV-related HCC in our cohort ‒ which is strongly associated with elevated AFP and DCP levels compared to NAFLD- or HCV-related HCC^14^^,^^22^ ‒ provides a more pronounced biomarker signal, thereby augmenting the model's discriminatory power. The underlying mechanism for these elevated biomarker levels in our population may be linked to the predominance of HBV genotypes B and C. Genotype C, in particular, is associated with more frequent BCP mutations and enhanced activation of oncogenic pathways, often resulting in poorer tumor differentiation and consequently greater AFP and DCP elevation.^23^^,^^24^ In contrast, NAFLD- or HCV-related HCC, more common in Western cohorts, typically exhibits lower AFP and DCP levels due to comparatively weaker reactivation of fetal gene programs and less disruption of vitamin K metabolism.^25^^,^^26^ Therefore, the efficacy of the GALAD model is not uniform but is context-dependent, modulated by regional variations in etiology, disease stage distribution, and control group composition.

External validation is critical to confirm the generalizability and adaptability of diagnostic models across diverse patient populations and clinical settings.^27^ By conducting external validation with local data, the authors aimed to demonstrate that GALAD is applicable and effective within our specific population. In the present study, the authors used the Hosmer-Lemeshow 10-group method to evaluate the model’s goodness of fit, with a resulting p-value greater than 0.05 indicating strong calibration in diagnosing Hepatocellular Carcinoma (HCC) among Chinese patients with HBV, primarily those infected with HBV (HBV). This approach underscores GALAD's potential utility as a reliable diagnostic tool in diverse HCC populations, including the local context, by confirming its diagnostic accuracy and adaptability.

Despite the GALAD score’s demonstrated efficacy, newer diagnostic scores and biomarkers have yet to be incorporated into clinical guidelines, with AFP and Ultrasound (US) remaining the primary recommended surveillance modalities. However, the US faces challenges related to accessibility and operator dependency, particularly in low-resource settings where trained sonographers are scarce. In this context, the GALAD score offers a simpler, more interpretable alternative or complementary tool. Notably, combining the GALAD score with US has shown exceptional diagnostic performance (sensitivity: 95 %; specificity: 91 %; AUC: 0.98) in HCC detection,^28^ In China, where HBV infection is widespread and HCC risk is high, particularly in underserved regions, such an approach may significantly improve early detection. To inform region-specific guidelines, large-scale, multi-center studies evaluating the combined use of the GALAD score and US are urgently needed. In light of the growing HBV-infected population and disparities in healthcare access, implementing this strategy could transform HCC diagnosis and reduce disease burden in China.

This study has several limitations. First, the patient cohort exclusively comprised individuals with hepatitis B, and detailed data on antiviral therapy regimens were not collected. As antiviral therapy is known to suppress baseline Alpha-Fetoprotein (AFP) levels and potentially enhance the diagnostic performance of AFP in surveillance testing, this factor may have influenced these findings. Future research should stratify patients by therapy status to clarify this impact. Second, the absence of detailed data on liver function reserve (e.g., Child-Pugh class or MELD score) limited their ability to adjust for its potential influence on biomarker levels such as DCP; subsequent studies should incorporate these variables as covariates. Third, the lack of HCC staging data restricted the evaluation of the GALAD score’s performance in early-stage disease, which is critical for screening programs. Future investigations should stratify by Barcelona Clinic Liver Cancer (BCLC) stage to assess sensitivity in early HCC, thereby enhancing clinical applicability. Finally, as this was a single-center study with a relatively limited sample size, the generalizability of the results may be constrained. In the future, the authors will collaborate with multiple centers to conduct larger validation studies.

## Conclusion

The GALAD model demonstrates high diagnostic accuracy for HBV-related HCC in the Chinese population. This cost-effective tool holds particular promise for tertiary prevention of HBV-related HCC, especially in resource-limited settings where access to advanced imaging modalities such as ultrasound and MRI remains critically constrained.

## Author's contribution

Keheng Li: Conceptualization, Methodology, Software, Visualization, Supervision, Writing-reviewing and editing.

Fei Xia: Data Collect, Visualization, Investigation, Investigation, Writing-original draft preparation.

## Ethics approval and consent to participate

This study was conducted in accordance with the Declaration of Helsinki and approved by the Ethics Committee of Ruian People's Hospital (Approval n° LZM2021056). Given the retrospective nature of the study and the use of anonymized data, the requirement for individual informed consent was waived by the Ethics Committee.

## Data availability statement

The datasets generated and/or analyzed during the current study are available from the corresponding author upon reasonable request.

## Conflicts of interest

The authors declare no conflicts of interest.
